# A novel prognostic signature and therapy guidance for hepatocellular carcinoma based on STEAP family

**DOI:** 10.1186/s12920-023-01789-0

**Published:** 2024-01-08

**Authors:** Dongxue Fu, Xian Zhang, Yi Zhou, Shanshan Hu

**Affiliations:** 1 Department of Anesthesiology, The First Affiliated Hospital of Wenzhou Medical University, Wenzhou, Zhejiang, China; 2 Key Laboratory of Diagnosis and Treatment of Severe Hepato-Pancreatic Diseases of Zhejiang Province, The First Affiliated Hospital of Wenzhou Medical University, No.1 South Baixiang Street, Ouhai District, Wenzhou, Zhejiang, 325000 China

**Keywords:** Hepatocellular carcinoma, STEAP, Prognosis signature, Immunotherapy, Drug sensitivity

## Abstract

**Background:**

The six-transmembrane epithelial antigen of prostate (STEAP) family members are known to be involved in various tumor-related biological processes and showed its huge potential role in tumor immunotherapy.

**Methods:**

Biological differences were investigated through Gene set enrichment analysis (GSEA) and tumor microenvironment analysis by CIBERSORT. Tumor mutation burden (TMB), immunotherapy response and chemotherapeutic drugs sensitivity were estimated in R.

**Results:**

We established a prognostic signature with the formula: risk score = STEAP1 × 0.3994 + STEAP4 × (− 0.7596), which had a favorable concordance with the prediction. The high-risk group were enriched in cell cycle and RNA and protein synthesis related pathways, while the low-risk group were enriched in complement and metabolic related pathways. And the risk score was significantly correlated with immune cell infiltration. Most notably, the patients in the low-risk group were characterized with increased TMB and decreased tumor immune dysfunction and exclusion (TIDE) score, indicating that these patients showed better immune checkpoint blockade response. Meanwhile, we found the patients with high-risk were more sensitive to some drugs related to cell cycle and apoptosis.

**Conclusions:**

The novel signature based on STEAPs may be effective indicators for predicting prognosis, and provides corresponding clinical treatment recommendations for HCC patients based on this classification.

**Supplementary Information:**

The online version contains supplementary material available at 10.1186/s12920-023-01789-0.

## Background

Hepatocellular carcinoma (HCC) is one of the most common cancers with high incidence and mortality [1, 2]. Although therapies have advanced, the mortality of HCC remains high due to the difficulty of diagnosis at early stage [3]. Moreover, it is generally observed that HCC is unfavorable response to radiation and chemotherapy, and the prognosis of patients who receive potentially curative treatment remains poor due to the high rate of recurrence. Therefore, it is of great significance to explore effective prognostic diagnostic models and treatment strategies for HCC.

The six-transmembrane epithelial antigen of prostate (STEAP) family of proteins are located on the cell surface, and it comprises 4 members, named STEAP1, STEAP2, STEAP3, and STEAP4. Cumulative evidence has revealed that STEAPs are abnormally expressed in various cancer tissues and cell lines [4, 5], and are significantly associated with patient prognosis [6, 7]. STEAPs can promote tumorgenesis and development through a variety of biological processess [8–13]. Studies have also shown that STEAP mRNA is detectable in serum of patients with different solid tumours [14], suggesting its potential as a tumor detection marker. Moreover, emerging studies have reported that STEAP is closely related to tumor immunity [15–18]. STEAP is a target of CD8+ T cells [19, 20], which renders STEAP an appealing candidate for tumor immunotherapy. In recent years, STEAP therapeutic peptides and STEAP vaccines [20–22] have been used as new methods for tumor treatment, and have been verified to show good effectiveness. Cappuccini et al. found that the combination of STEAP1 vaccine and PD-1 blocking antibody can significantly improved survival of the animals, with 80% of mice remaining tumor-free [23]. Sebastian et al. found that there is a strong antitumor potential of MHC class I-restricted TCR-transgenic CD4+ T cells against a STEAP1-derived peptide in vivo [24]. However, systematic analysis of STEAPs expression profile and function in HCC were still insufficient. Given that, further insight into the function and the role of STEAPs in HCC may provide novel approach for precise treatment and individualized management.

## Methods

### Study subjects

The RNA-seq profiles and clinical data of HCC patients and normal samples were acquired from The Cancer Genome Atlas (TCGA, http://cancergenome.nih.gov/), and GSE14520 data set stored in the Gene Expression Omnibus database (GEO, https://www.ncbi.nlm.nih.gov/geo/). The TCGA was used as the training set, and contained 371 tumor samples, 50 normal samples, while the GSE14520 as the validation sets and contained 244 tumor samples.

### Identification of differentially expressed and prognostic-related STEAPs

The differentially expressed STEAPs were identified by Student’s two-tailed t-test in R 4.0.3. Univariate Cox proportional hazard regression analysis was used to screen the prognostic-related STEAPs. The median survival time and cumulative survival curves were determined by the Kaplan–Meier method. Then, STEAPs with both *P* ≤ 0.05 were further identified by multivariate Cox proportional hazard regression. Genes with *P* ≤ 0.05 in multivariate Cox proportional hazard regression analysis was identified as prognostic-related STEAPs for further analysis.

### Development and assessment of STEAPs-based prognostic risk model

Risk scores were established using the gene expression values and its prognostic weight coefficients that calculated by the multivariate Cox proportional hazard regression analysis with the following formula:

$$\textrm{risk}\ \textrm{score}={\sum}_{i=1}^n{\beta}_i\times {Exp}_i$$

Based on the formula, the risk score of each patient was calculated, and patients were subdivided into high- or low-risk groups according to the median levels of the risk score. The cumulative survival curves of the grouped patients were determined by the Kaplan–Meier and differences between the groups were analyzed using the log-rank test. *P* ≤ 0.05 was considered statistically significant. The prognostic performance of the risk score model was assessed by using receiver operating characteristic (ROC) curve analysis within 0.5, 1, and 3 years. Univariate and multivariate Cox hazard regression was used to assess the impact of some prognostic factors.

### Building and validation of the nomogram

The clinical characters consisting of age, gender, stage, liver fibrosis, and risk score *et al.* were selected to construct a prognostic nomogram to help predict the probability of 1-, 3-, and 5-year overall survival rates for HCC patients via the rms R package. The prediction power of the nomogram was graphically displayed via calibration curve.

### Gene set enrichment analysis and tumor microenvironment analysis

Differentially expressed genes (DEGs) among the low- and high-risk groups were determined with Student’s two-tailed t-test. Genes with a *P* ≤ 0.05 and |log2FoldChange| ≥ 1 were defined as a differential gene. Gene set enrichment analysis (GSEA) analysis was implemented to determine the functional pathways enriched by high- and low-risk groups. The KEGG gene set (c2.cp.kegg.v7.0.symbols.gmt) was derived from the website (https://www.gsea-msigdb.org/). The expression of immune cells were evaluated by CIBERSORT algorithm.

### Tumor mutation profile, immunotherapy response prediction, and therapeutic drug sensitivity

The original mutation annotation format of each LIHC sample was acquired from TCGA. Then, we calculated the tumor mutation burden (TMB) score according to the somatic mutation data and computed the differences of TMB between the low- and high-risk groups. The analyses were based on R package “maftools”. Subsequently, tumor immune dysfunction and exclusion (TIDE) database (http://tide.dfci.harvard.edu/) were applied to predict the potential immune checkpoint blockade (ICB) response. Finally, we used R package “pRRophetic” to calculate the semi-inhibitory concentration (IC50) values of chemotherapeutic drugs.

## Results

### Aberrant expression of STEAPs in HCC samples

**Fig. 1:**
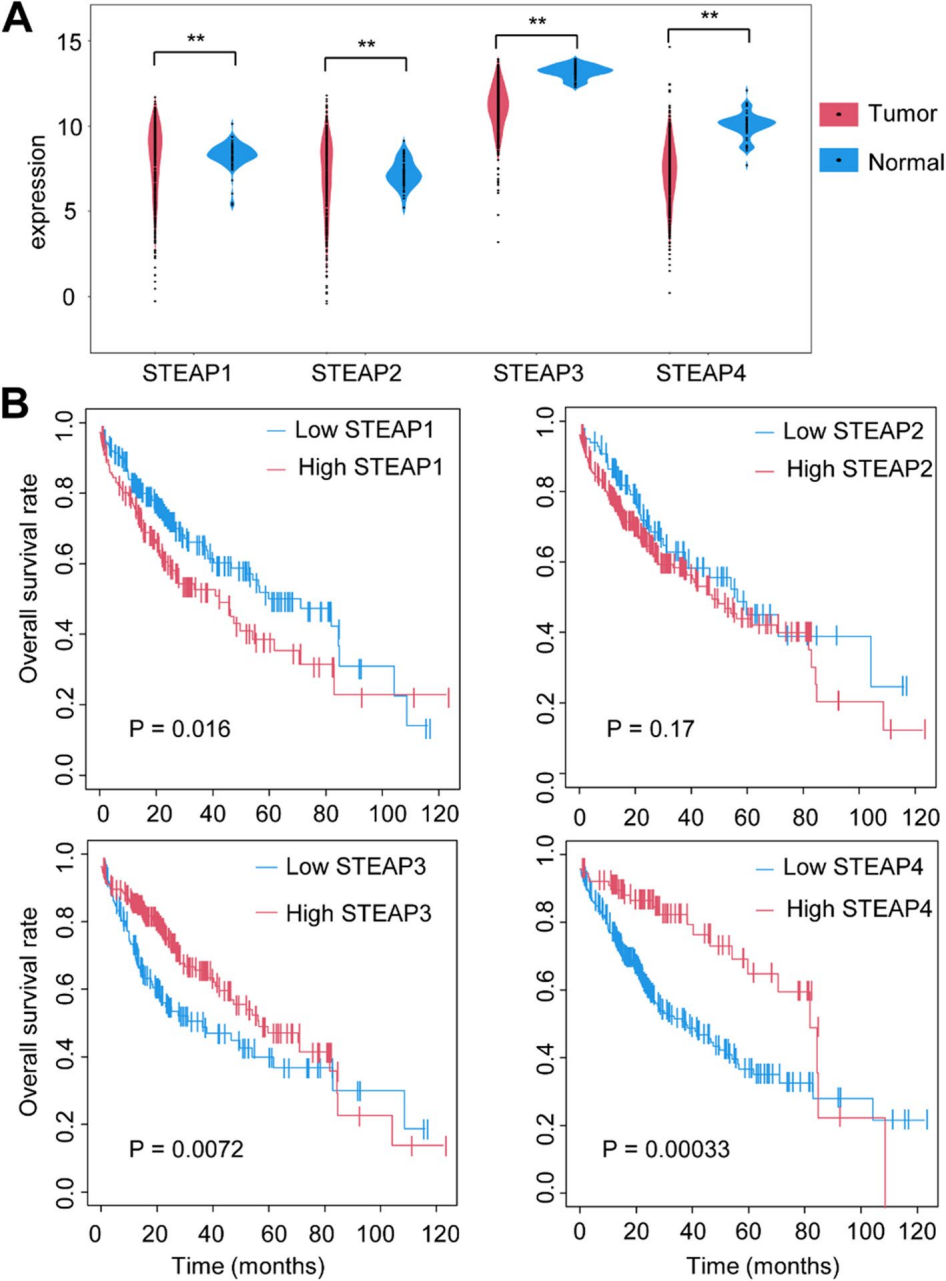
Identification of differentially expressed and prognostic-related STEAPs in the TCGA cohorts. **A** Violin plots showed the expression profile of STEAP family in HCC tumor tissues and normal tissues. **B** Survival analysis of STEAP family related signatures. **P < 0.01

Based on data from TCGA cohort, expression levels of STEAP1 (*P* = 9.22E-05) and STEAP2 (*P* = 7.33E-07) were significantly increased, while STEAP3 (*P* = 6.73E-26) and STEAP4 (*P* = 7.91E-09) was significantly decreased in tumors compared with adjacent normal tissues (Fig. 1A).

### Identification of two prognostic-related STEAPs in HCC samples

In order to further find the prognostic-related STEAPs, we analyzed the relationship between the expression level of STEAPs and overall survival (OS) rate of HCC patients. Following univariate Cox regression analysis, STEAP1, STEAP3, and STEAP4 were obtained for exhibiting significant prognostic correlation with OS (Fig. 1B, Table 1). In addition, Kaplan–Meier survival curves based on the median expression value indicated that the lower expression of STEAP1 had a better prognosis in terms of OS (*P* = 0.016), while the higher expression of STEAP3 and STEAP4 showed a better prognosis (*P* = 0.0072 and 0.00033, respectively; Fig. 1B). Subsequently, multivariate Cox regression analysis indicated that STEAP1 (hazard ratio [HR]: 1.49; *P* = 0.024) and STEAP4 (HR: 0.47; *P* = 0.0031) exhibited independent prognostic value for HCC (Table 1).

**Table 1:** Cox analysis of STEAP family in the TCGA cohort

Gene	Univariate Cox analysis				Multivariate Cox analysis			
	HR	Low 95% CI	High 95% CI	*P*	HR	Low 95% CI	High 95% CI	*P*
STEAP1	**1.53**	**1.08**	**2.16**	**0.016**	**1.49**	**1.06**	**2.11**	**0.024**
STEAP2	**1.32**	**0.89**	**1.96**	**0.17**	**–**	**–**	**–**	**–**
STEAP3	**0.62**	**0.44**	**0.88**	**0.0072**	**0.72**	**0.51**	**1.03**	**0.071**
STEAP4	**0.41**	**0.25**	**0.67**	**0.00033**	**0.47**	**0.28**	**0.77**	**0.0031**

### Construction and validation of the STEAP1 and STEAP4 based prognostic risk score

**Fig. 2:**
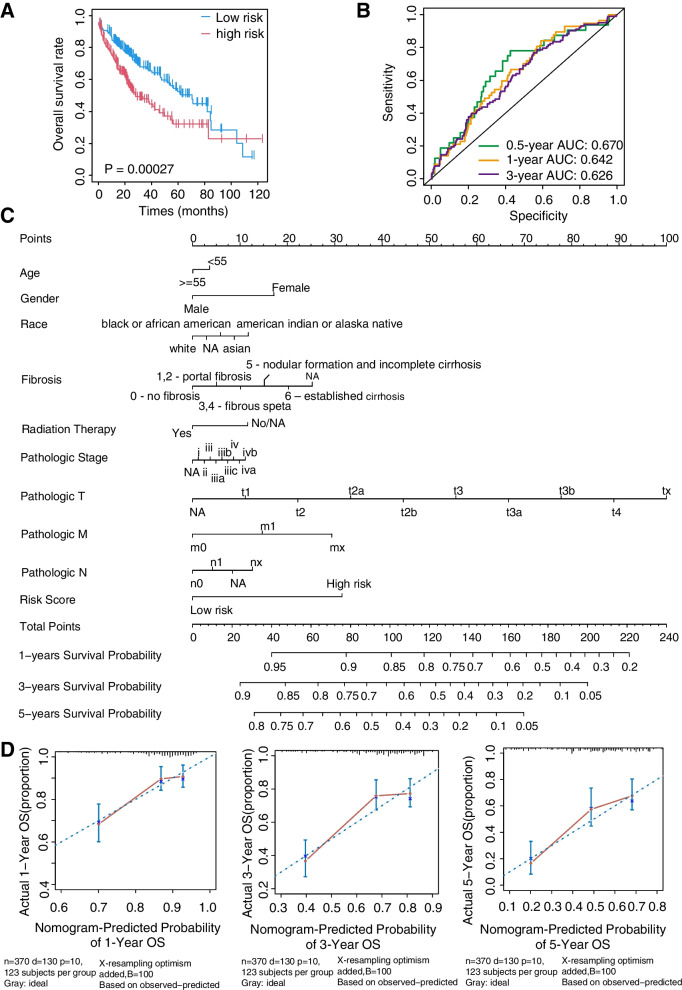
The validation of the prognostic risk model and the nomogram and calibration curve of the model in the TCGA cohorts. **A** Kaplan–Meier survival curves based on risk score. **B** 0.5-, 1-, and 3-year ROC curves based on risk score. **C** Nomogram for predicting overall survival. **D** The calibration curves for 1-, 3-, and 5-year OS

The two prognostic-related genes STEAP1 and STEAP4 were selected to establish a risk score model with the formula as follows: risk score = STEAP1 × 0.3994 + STEAP4 × (− 0.7596). Then, we analyzed the distribution of risk scores (Additional file 1: Fig. S1) and divided all HCC patients in the training and validation groups into high-risk and low-risk groups based on the median risk score. To evaluate the effectiveness of STEAPs-based prognostic risk model, we analyzed the prognosis of the two grouped patients and found that OS of HCC patients in the low-risk group was significantly better than that in the high-risk group in the TCGA training (*P* = 0.00027; Fig. 2A) and GSE14520 validation cohorts (*P* = 0.030; Additional file 1: Fig. S2A). Moreover, ROC curve analysis showed that the risk score model had the favorable predictive ability of the 0.5, 1 and 3 year OS, with an area under the ROC curve (AUC) of 0.670, 0.642, and 0.626 in the training cohort (Fig. 2B), and with AUC of 0.641, 0.635, and 0.534 in the validation cohort (Additional file 1: Fig. S2B).

### Construction of nomogram for prognosis evaluation

To verify the independence of the risk score model, we conducted univariate and multivariate COX analysis on the demographic characteristics of all patients in the TCGA and GSE14520 cohorts. The result showed that the risk score was significantly correlated with OS in the TCGA training cohort (HR = 1.89/1.81, *p* < 0.001) and GSE14520 validation cohort (HR = 1.56/1.27, *p* = 0.031/0.29; Table 2). Furthermore, the demographic characteristics including risk score were enrolled to construct a nomogram for predicting the 1-, 3-, and 5-year OS incidences (Fig. 2C, Additional file 1: Fig. S2C). The calibration curves exhibited a favorable consistency with the actual observation (Fig. 2D, Additional file 1: Fig. S2D).

**Table 2:** Clinical characters of HCC patients in the TCGA training cohort and GSE14520 validation cohorts

Set	Factors	Univariate Cox analysis		Multivariate Cox analysis	
		HR(95% CI)	*P*	HR(95% CI)	*P*
**TCGA (training set)**	Age	1.24 (0.88–1.76)	0.23	1.14 (0.76–1.70)	0.53
	Gender	1.22 (0.86–1.73)	0.28	1.23 (0.85–1.78)	0.072
	Race	0.87 (0.72–1.05)	0.14	1.22 (0.94–1.58)	0.14
	Pathologic_stage	1.64 (1.34–2.00)	**1.81E-06**	1.14 (0.50–2.62)	0.75
	Pathology_T_stage	1.61 (1.36–1.91)	**2.94E-08**	1.46 (0.66–3.21)	0.35
	Pathology_N_stage	1.25 (1.04–1.50)	**0.017**	1.07 (0.81–1.42)	0.63
	Pathology_M_stage	1.29 (1.07–1.55)	**0.0068**	1.42 (1.06–1.89)	**0.018**
	Risk score	1.89 (1.34–2.69)	**3.41E-04**	1.81 (1.21–2.70)	**0.0037**
**GSE14520 (validation set)**	Age	0.80 (0.53–1.19)	0.26	1.05 (0.67–1.66)	0.82
	Gender	0.54 (0.26–1.11)	0.093	0.81 (0.39–1.71)	0.58
	TNM_staging	2.34 (1.77–3.09)	**2.18E-09**	1.42 (0.98–2.06)	0.067
	BCLC_staging	2.22 (1.75–2.81)	**3.34E-11**	1.36 (0.91–2.03)	0.13
	CLIP_staging	1.92 (1.55–2.38)	**1.65E-09**	1.45 (0.93–2.24)	0.099
	AFP	1.69 (1.13–2.53)	**0.011**	0.84 (0.43–1.64)	0.61
	Risk score	1.56 (1.04–2.33)	**0.031**	1.27 (0.82–1.97)	0.29

### Analysis of DEGs and their functional pathways of the prognostic risk groups

**Fig. 3:**
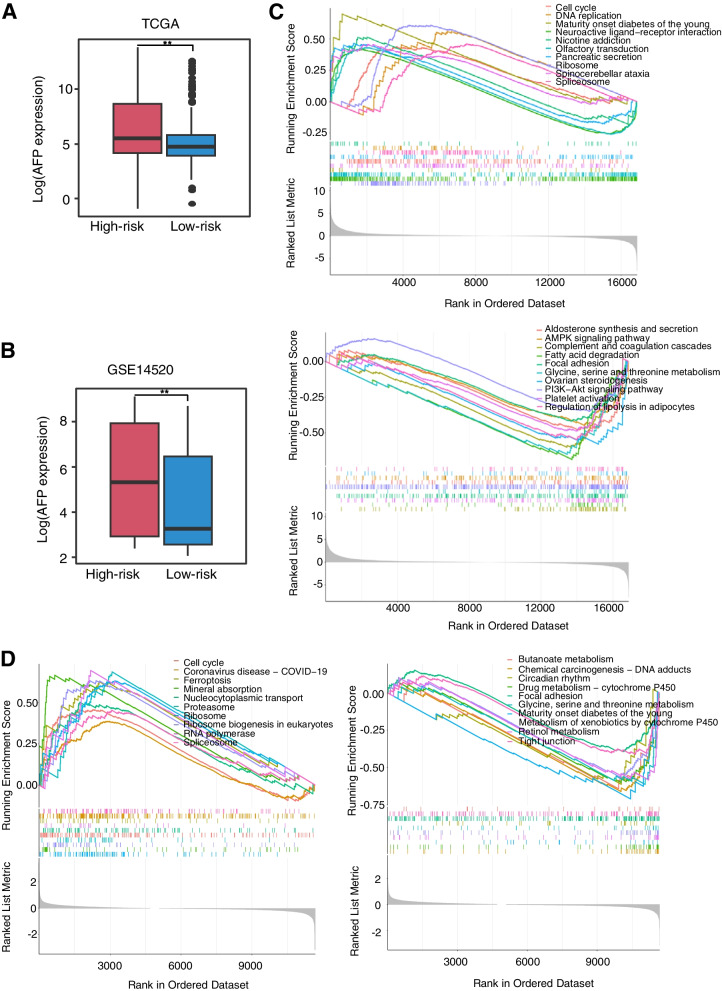
Differential expression of AFP between two groups and functional enrichment analysis of the two groups. **A**,** B** Boxplot showed that the expression of AFP at high-risk group is significantly higher than that at low-risk group in the (**A**) TCGA and (**B**) GSE14520 cohorts. **C, D** The GSEA analysis for TCGA (**C)** and GSE14520 (**D**). **P < 0.01

A total of 1119 DEGs were identified in TCGA, including 564 upregulated and 555 downregulated (Additional file 2: Table S1). Simultaneously, GSE14520 confirmed 39 DEGs, with 17 upregulated and 22 downregulated (Additional file 3: Table S2). Among them, the expression of AFP——an indicator often used for diagnosis of HCC, showed very significant differences between the high-risk group and the low-risk group both in the training set (*P* = 0.0041, OR = 4.49) and the validation sets (*P* = 0.00029, OR = 2.35; Fig. 3A, B). Then, we performed GSEA analyses to further investigate functional pathways associated with the prognostic risk groups. The results showed that genes in the high-risk group were enriched in the pathways of cell cycle and RNA and protein synthesis related pathways (ribosome, spliceosome, DNA replication, proteasome, etc). However, genes in the low-risk group were enriched in the complement and coagulation cascade pathways, as well as pathways related to amino acid, fatty acid, and drug metabolism (Fig. 3C, D, Additional file 4: Table S3, Additional file 5: Table S4).

### Investigation of the immune microenvironment in risk groups

**Fig. 4:**
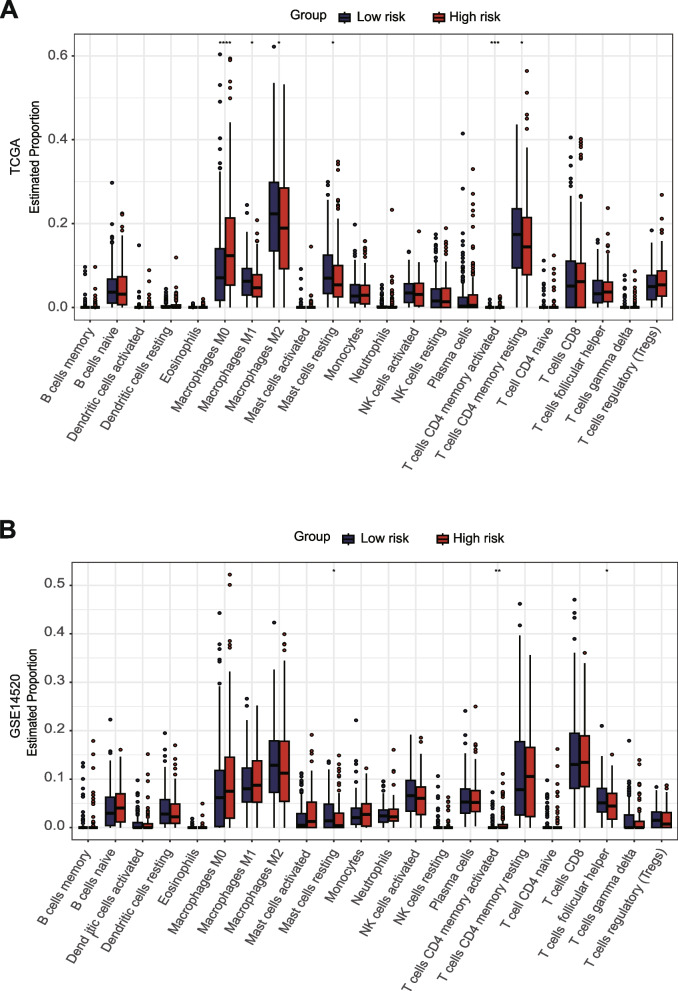
Immune signature in two groups. **A, B** discrepancy analysis of tumor-infiltrating immune cells between two groups in the (**A**) TCGA and (**B**) GSE14520 cohorts

To explore the immune microenvironment in the prognostic risk groups, we calculated the expression of the tumor-infiltrating immune cell between two risk groups. Patients in the high-risk exhibited a significant decrease in Macrophages M1, Macrophages M2, Mast cells resting, T cells CD4 memory resting and a significant increase in Macrophages M0, T cells CD4 memory activated (Fig. 4A, B).

### TMB, TIDE and therapeutic drug sensitivity

**Fig. 5:**
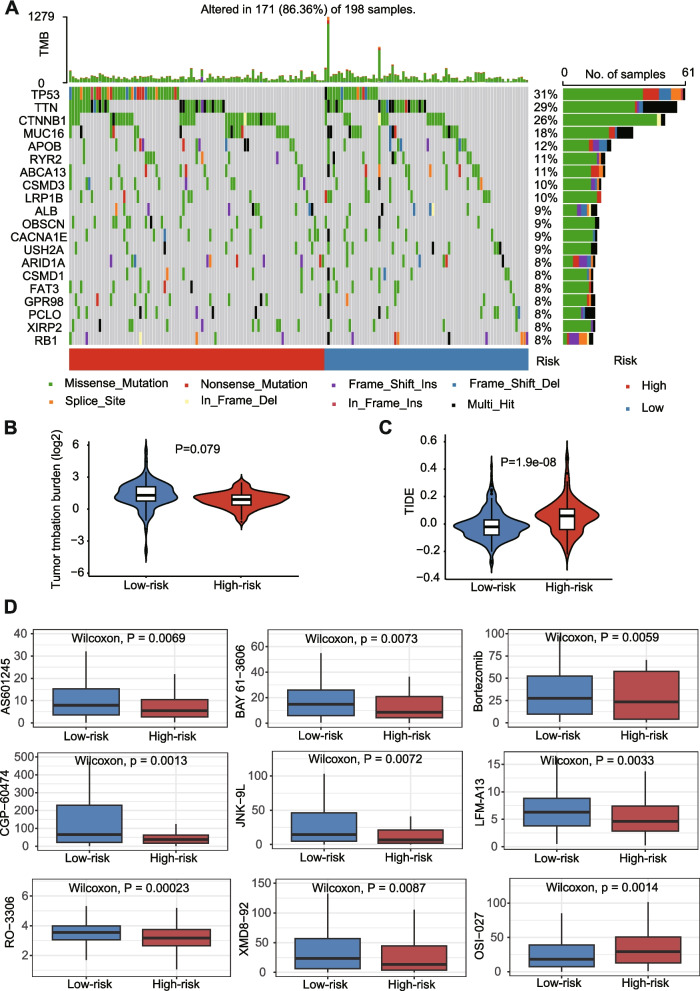
TMB analysis, immunotherapy response, and therapeutic drug sensitivity prediction. **A** OncoPrint of frequently mutated genes in high- and low-risk groups. **B** TMB difference between high- and low-risk groups. **C** TIDE score between two groups. **D** chemotherapeutic drugs with significant IC50 differences between the two groups

Then, we analyzed the variations of the somatic mutations in two risk groups. The highest mutated genes were TP53, TTN, CTNNB1, MUC16, APOB, RYR2, ABCA13, CSMD3, and LRP1B (Fig. 5A). Compared with high-risk group, patients in low-risk group had higher TMB (Fig. 5B). The TIDE score was significantly higher in high-risk group compared with low-risk group (*P* = 1.90E-08; Fig. 5C). Through drug sensitivity comparison, we found that patients in high-risk group were more sensitive to AS601245, BAY 61–3606, Bortezomib, CGP − 60,474, JNK − 9 L, LFM-A13, RO − 3306, and XMD8–92, while patients in low-risk group were more sensitive to OSI − 027 (Fig. 5D).

## Discussion

STEAPs are unique to mammals and were initially identified as important metalloreductases in vivo [25]. They are involved in a wide range of biological processes, such as molecular trafficking in the endocytic and exocytic pathways and control of cell proliferation and apoptosis [26, 27]. Lots of studies have shown that STEAPs are abnormally expressed in a variety of human cancers and play vital role in promoting tumorgenesis and development [28–30], making them become potential prognostic biomarker, detection biomarker and therapeutic target [7, 14, 31], However, the clinical significance and role of STEAPs in HCC are still unclear.

In this study, we screened the differentially expressed and prognostic-related STEAPs in the TCGA training set and confirmed two significantly prognostic-related genes (STEAP1 and STEAP4), whose roles in HCC were barely studied. STEAP1 plays an important role in intercellular communication, modulating the transport of small molecules and ions such as Na+, K+ and Ca2+, and releasing soluble cytokines and chemokines [8, 32]. Moreover, STEAP1 is highly expressed in multiple cancer tissues such as prostate, bladder, ovarian, and colon cancer and has the role of promoting invasion of tumor cells [4, 33–35]. Several studies have showed that its overexpression inhibits apoptosis and induces epithelial to mesenchymal transition, ultimately contributing to tumor progression and aggressiveness in cancer cells [36–38]. In addition, STEAP1 has been considered as an optimal target for T cell-based immunotherapy, with applications in a subset of cancer types nowadays [5, 39]. STEAP4 is also called STAMP2 and TNF-α induce adipose-related protein (TIARP), which can regulate inflammatory reaction, fatty acid metabolism and glucose metabolism [40–42]. Besides, studies have demonstrated that STEAP4 is also closely related to tumorigenesis [43–45].

Subsequently, we established and validated a risk score model based on the expression of STEAP1 and STEAP4, and divided patients into low- and high-risk groups according to the median values. Studies have shown that certain transcription factors responsible for the holistic progression of fibrosis in HCC are transcriptional regulators of STEAP1 and STEAP4 [46]. To further explore the association between STEAP1 and STEAP4 with the development of HCC, we analyzed the correlation between their expression with some particular etiology (alcohol consumption, hepatitis, non-alcoholic fatty liver disease) and liver fibrosis (including cirrhosis) in HCC. The results showed that STEAP1, STEAP4, and risk score were not correlated with specific etiology, while STEAP1 and risk score were significantly positively correlated with liver fibrosis both in TCGA and GSE14520 (Additional file 1: Fig. S3). These results suggest that STEAP1 and our risk score may have a potentially important role in the progression of liver fibrosis to HCC, but not in the process from pathology to liver fibrosis, which needs to be verified by further experiments.

Numerous studies have shown that immune checkpoint inhibitors are far more effective than chemotherapy in tumor patients with high TMB expression [47, 48]. TIDE algorithm is a method for predicting ICB response in cancer. A higher TIDE score is associated with worse ICB response. Our results suggest that patients in the low-risk group can receive better benefits from clinical immunotherapy. Meanwhile, we used pRRophetic to calculate the IC50 values of chemotherapeutic drugs, and found that patients in high-risk group were more sensitive to AS601245, BAY 61–3606, Bortezomib, CGP − 60,474, JNK − 9 L, LFM-A13, RO − 3306, and XMD8–92. Among them, AS601245, JNK-9 L, and LFM-A13 are selective inhibitors of c-jun-N-terminal kinase (JNK). BAY 61–3606 is an orally available, ATP-competitive, reversible and highly selective Syk inhibitor. Bortezomib is a reversible and selective proteasome inhibitor that effectively inhibits the 20S proteasome by targeting threonine residues. CGP60474 and RO-3306 are potent cyclin-dependent kinase (CDK) inhibitors. XMD8–92 is a potent ERK5 (BMK1)/BRD4 inhibitor. This result shown that all drugs sensitive to high-risk groups with anti-cancer activity by affecting cell cycle and inducing apoptosis [49–55], which coincides with our result of pathway enrichment in the high-risk group. Our study may provide corresponding clinical treatment recommendations for HCC patients based on this classification.

In the present study, there still exist some limitations. First, the prognostic signature was created and verified based on retrospective data from TCGA and GSE14520 databases. Further large scale prospective clinical studies are required to evaluate its effectiveness and practicability. Besides, more well-designed basic research experiments are warranted to highlight the crucial role of STEAPs and corresponding treatment strategies in the precise treatment of HCC.

## Conclusions

Current research indicates that novel signature based on STEAPs may be effective indicators for predicting prognosis, and provides corresponding clinical treatment recommendations for HCC patients based on this classification.

### Supplementary Information

**Additional file 1: Fig. S1.** The histogram of the distribution of risk scores in the TCGA and GSE14520. **Fig. S2.** The validation of the prognostic risk model and the nomogram and calibration curve of the model in the GSE14520 cohorts. **Fig. S3.** The correlation between the expression of STEAP1, STEAP4 and risk score with particular etiology and liver fibrosis in the TCGA and GSE14520.

**Additional file 2: Table S1. **Differentially expressed genes of the two prognostic risk group in the TCGA.

**Additional file 3: Table S2. **Differentially expressed genes of the two prognostic risk group in the GSE14520.

**Additional file 4: Table S3. **The GSEA analysis for TCGA.

**Additional file 5: Table S4. **The GSEA analysis for GSE14520.

## Data Availability

All data used in this study can be acquired from TCGA (http://cancergenome.nih.gov/) and GSE14520 stored in the GEO (https://www.ncbi.nlm.nih.gov/geo) databases.

## Abbreviations

STEAP: Six-transmembrane epithelial antigen of prostate
TMB: Tumor mutation burden
TIDE: Tumor immune dysfunction and exclusion
HCC: Hepatocellular carcinoma
TCGA: The Cancer Genome Atlas
OS: Overall survival
GSEA: Gene set enrichment analysis
GEO: Gene Expression Omnibus database
ROC: Receiver operating characteristic
DEGs: Differentially expressed genes
ICB: Immune checkpoint blockade
IC50: Semi-inhibitory concentration
