# Supplementary material for: A novel prognostic signature and therapy guidance for hepatocellular carcinoma based on STEAP family
Source: BMC Med Genomics. 2024 Jan 8;17:16. doi: 10.1186/s12920-023-01789-0 (PMC10775544; doi:10.1186/s12920-023-01789-0)
Supplement: Supplementary file 1 — Additional file 1: Fig. S1. The histogram of the distribution of risk scores in the TCGA and GSE14520. Fig. S2. The validation of the prognostic risk model and the nomogram and calibration curve of the model in the GSE14520 cohorts. Fig. S3. The correlation between the expression of STEAP1, STEAP4 and risk score with particular etiology and liver fibrosis in the TCGA and GSE14520. [file 12920_2023_1789_MOESM1_ESM.docx]

**
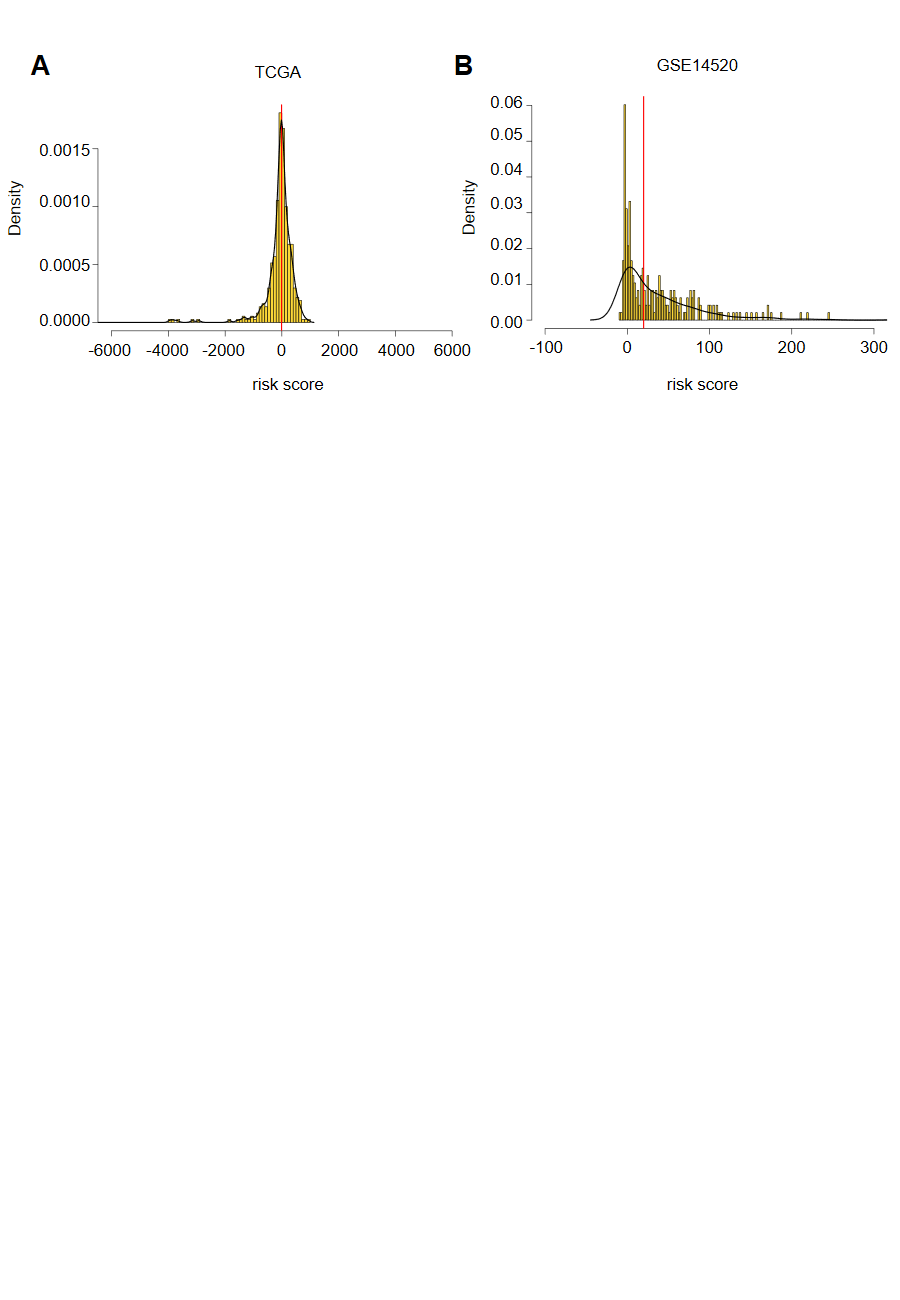
**

**Fig. S1. The histogram of the distribution of risk scores in the TCGA and GSE14520.**


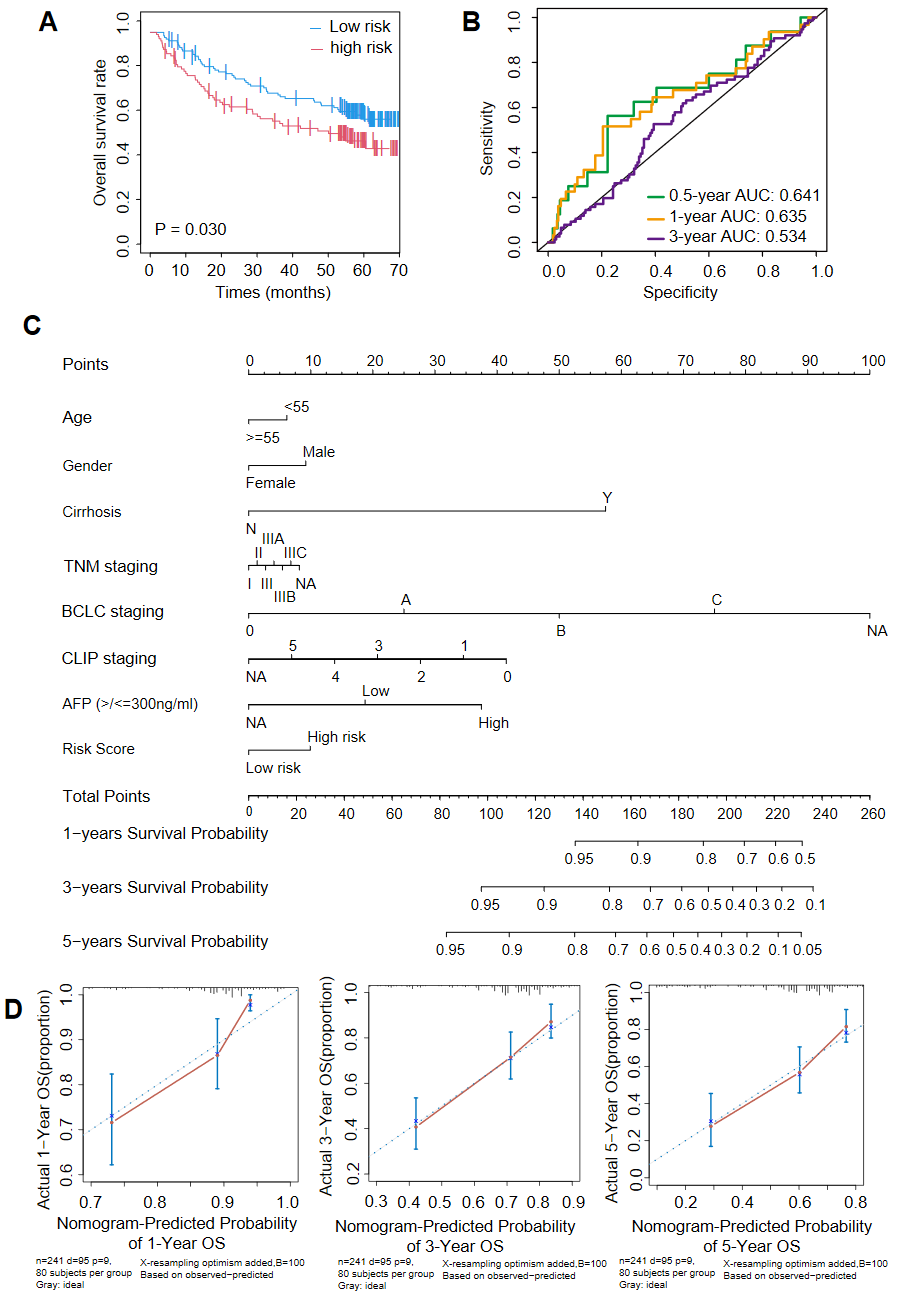


**Fig. S2. The validation of the prognostic risk model and the nomogram and calibration curve of the model in the GSE14520 cohorts.** (**A**) Kaplan–Meier survival curves based on risk score. (**B**) 0.5-, 1-, and 3-year ROC curves based on risk score. (**C**) Nomogram for predicting overall survival. (**D**) The calibration curves for 1-, 3-, and 5-year OS.


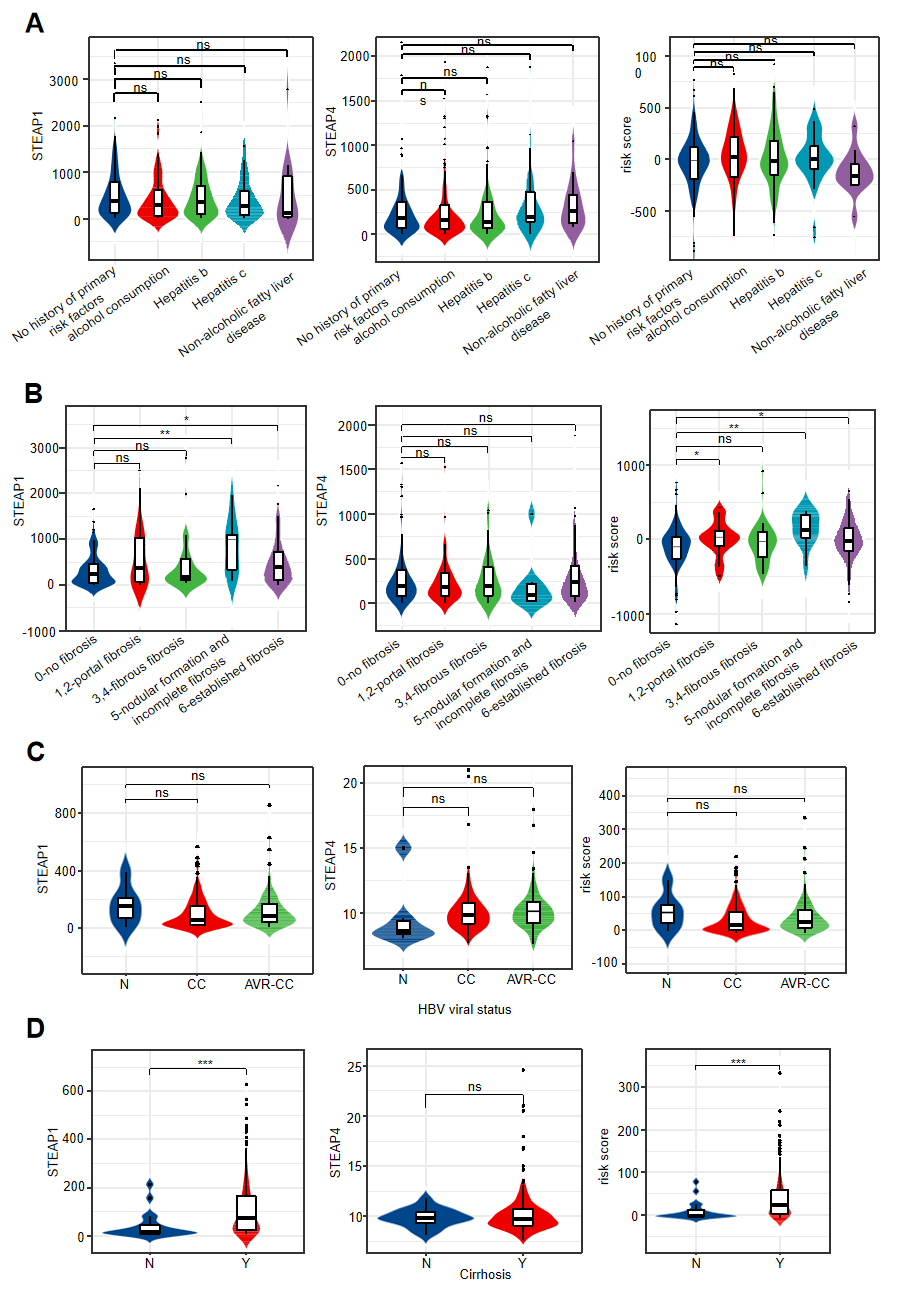


**Additional fle 1: Fig. S3.** **The correlation between the expression of STEAP1, STEAP4 and risk score with particular etiology and liver fibrosis in the TCGA and GSE14520**. (**A**) The correlation between their expression with alcohol consumption, hepatitis b, hepatitis c, non-alcoholic fatty liver disease in the TCGA. (**B**) The correlation between their expression with liver fibrosis in the TCGA. (**C**) The correlation between their expression with HBV virus status in the GSE14520. (**D**) The correlation between their expression with cirrhosis in the GSE14520.
